# The mechanism and application of traditional Chinese medicine extracts in the treatment of lung cancer and other lung-related diseases

**DOI:** 10.3389/fphar.2023.1330518

**Published:** 2023-12-06

**Authors:** Zhenglin He, Yihan Wang, Liang Han, Yue Hu, Xianling Cong

**Affiliations:** ^1^ China-Japan Union Hospital of Jilin University, Jilin University, Changchun, China; ^2^ Department of Pathology, China-Japan Union Hospital of Jilin University, Changchun, China; ^3^ Department of Biobank, China-Japan Union Hospital of Jilin University, Changchun, China; ^4^ Department of Dermatology, China-Japan Union Hospital of Jilin University, Changchun, China

**Keywords:** Chinese medicine extracts, non-small cell lung cancer, small cell lung cancer, pulmonary fibrosis, acute lung injury, treatment

## Abstract

Lung cancer stands as one of the most prevalent malignancies worldwide, bearing the highest morbidity and mortality rates among all malignant tumors. The treatment of lung cancer primarily encompasses surgical procedures, radiotherapy, and chemotherapy, which are fraught with significant side effects, unfavorable prognoses, and a heightened risk of metastasis and relapse. Although targeted therapy and immunotherapy have gradually gained prominence in lung cancer treatment, diversifying the array of available methods, the overall recovery and survival rates for lung cancer patients remain suboptimal. Presently, with a holistic approach and a focus on syndrome differentiation and treatment, Traditional Chinese Medicine (TCM) has emerged as a pivotal player in the prognosis of cancer patients. TCM possesses characteristics such as targeting multiple aspects, addressing a wide range of concerns, and minimizing toxic side effects. Research demonstrates that Traditional Chinese Medicine can significantly contribute to the treatment or serve as an adjunct to chemotherapy for lung cancer and other lung-related diseases. This is achieved through mechanisms like inhibiting tumor cell proliferation, inducing tumor cell apoptosis, suppressing tumor angiogenesis, influencing the cellular microenvironment, regulating immune system function, impacting signal transduction pathways, and reversing multidrug resistance in tumor cells. In this article, we offer an overview of the advancements in research concerning Traditional Chinese Medicine extracts for the treatment or adjunctive chemotherapy of lung cancer and other lung-related conditions. Furthermore, we delve into the challenges that Traditional Chinese Medicine extracts face in lung cancer treatment, laying the foundation for the development of diagnostic, prognostic, and therapeutic targets.

## 1 Introduction

Primary bronchogenic lung cancer, commonly referred to as lung cancer, represents the most prevalent form of malignant tumor. It encompasses two primary subtypes: small cell lung cancer (SCLC) and non-small cell lung cancer (NSCLC), with NSCLC constituting a majority, accounting for 85%–90% of cases (Goldstraw et al., 2007). As per data from the 2020 World Cancer Report (GLOBOCAN), the global incidence of lung cancer surged to 2.2 million new cases, contributing to 11.4% of all newly reported cancer cases. Alarmingly, the number of fatalities reached 1.8 million, amounting to 18.0% of total cancer-related deaths. Both the incidence and mortality of this malignancy have consistently held the top position among malignant tumors, displaying a worrisome upward trajectory year after year (Li et al., 2023). Despite the armamentarium of Western medical interventions that encompass comprehensive approaches such as surgery, chemotherapy, radiation therapy, and molecular targeting, a staggering 80% of lung cancer patients already find themselves in the intermediate to advanced stages of the disease. Consequently, their 5-year survival rate remains dishearteningly low, at a mere 14%. Furthermore, these therapies are accompanied by significant side effects. A cascade of common toxic reactions during the chemotherapy process, including anemia, neutropenia, mucositis, or isopermeability, and special toxicity such as ototoxicity, nephrotoxicity, pulmonary toxicity, neurotoxicity, or Raynaud’s syndrome (Zraik and Heß-Busch, 2021). Radiotherapy also bears noticeable side effects, such as lung injury may cause radiation pneumonia, acute respiratory distress syndrome, bronchiolitis obliterans with mechanical pneumonia, and other diseases (Latrèche et al., 2022). While targeted therapy stands out for its precision and reduced side effects compared to other modalities, it is not without its challenges, notably the potential to cause hypertension, anemia, fever, and acute phase reactions (Tímár and Uhlyarik, 2022). With the deepening exploration of traditional Chinese medicine, multiple studies have unveiled its potential to profoundly enhance the quality of life for patients and extend their survival. In clinical applications, traditional Chinese medicine has demonstrated its capacity to inhibit and eliminate tumor cells, while also alleviating the adverse reactions associated with radiotherapy and chemotherapy.

## 2 Treatment of lung cancer with traditional Chinese medicine extract

### 2.1 The mechanism of traditional Chinese medicine extract treatment for non-small cell lung cancer

#### 2.1.1 Cell proliferation

Deregulated cell cycle progression stands as a pivotal catalyst for unbridled cell proliferation, ultimately culminating in the malignant transformation characteristic of cancer. A cornerstone in the quest to combat tumors lies in the inhibition of tumor cell proliferation. Traditional Chinese medicine extracts have emerged as a promising avenue for regulating the cell cycle by modulating the expression of genes integral to tumor proliferation, including cyclin, P53, PCNA, and reactive oxygen species, among others. For instance, a study by Zhu et al. (Zhu et al., 2021) unveiled the potential of licorice extract to curtail the growth of NSCLC. This effect was achieved by downregulating the expression of the CDK4-Cyclin complex, thus obstructing the G0/G1 phase progression in tumor cells. Furthermore, the compound resibufogenin, extracted from Venenum Bufonis, was found to activate GSK-3β in human lung cancer A549 cells, leading to the downregulation of cyclin D1 expression and instigating a G1 phase blockade (Ichikawa et al., 2015). Homoisoflavanone-1, isolated from polygonatum odoratum, exhibited notable prowess in inhibiting NSCLC growth and inducing apoptosis in a dose-dependent manner. This compound primarily arrested the cell cycle in the G2/M phase by activating the p38/p53 signaling pathway. Moreover, it spurred apoptosis in A549 cells through the regulation of mitochondrial/cysteine protease dependence and endoplasmic reticulum stress pathways (Ning et al., 2018). Salvia miltiorrhiza, specifically its methanol extract, tanshinone (CTN), demonstrated the capacity to induce cell cycle arrest in the G2/M phase. This effect was accompanied by an elevation in the expression of p53 and p21, as well as the activation of caspase-3/9 and PARP1. The net result was a pronounced inhibition of cell proliferation and the facilitation of early and late tumor cell apoptosis (Ye et al., 2017). Another extract from Salvia miltiorrhiza, tanshinone IIA, exhibited the ability to induce cell cycle arrest in the G1/S phase, impeding the progression of lung adenocarcinoma. This was achieved through the substantial downregulation of CCNA2, CDK2, AURKA, PLK1, and p-ERK expression (Li et al., 2021). Dioscin, sourced from Rhizoma Dioscoreae Nipponicae, wielded the power to significantly curtail the expression of p-AKT, MMP2, and PCNA, thereby inhibiting the *in vitro* proliferation, invasion, and migration of lung cancer cells. Additionally, it manifested the capability to reduce lung nodules, mitigate lung injury, and diminish mortality in mouse lung cancer models (Xi et al., 2022). The aqueous extract derived from Hedyotis diffusa, combined with Scutellaria barbata in an equal weight ratio (HDSB11), effectively diminished the expression of NLRP3, procapsase-1, caspase-1, PRAP, Bcl-2, and cyclin D1. This was accompanied by a reduction in the phosphorylation levels of NF-κB, ERK, JNK, and p38 MAPK, resulting in the inhibition of tumor cell proliferation (Lv et al., 2021). Hence, the multitude of traditional Chinese medicine monomers and extracts offer promise in the inhibition of tumor cell proliferation, cell cycle regulation, and the effective treatment of NSCLC.

#### 2.1.2 Cell apoptosis

Apoptosis, known as programmed cell death (PCD), is a meticulously orchestrated process of active cell demise initiated by shifts in the body’s internal and external environment or by death signals orchestrated through gene regulation. Tumors represent a complex spectrum of disorders characterized not only by abnormal cell proliferation and differentiation but also by irregular cell apoptosis. Recent research has unearthed compelling evidence regarding the impact of specific compounds on tumor cells. For instance, imperatorin derived from Angelica dahurica has been shown to modulate gene expression, upregulating p53 and Bax while simultaneously downregulating Mcl-1. This concerted action exerts a robust inhibitory effect on the autonomous growth of lung cancer cells (Choochuay et al., 2013). Ethyl acetate extract from Selaginella doederleinii Hieron intervenes in the cell cycle, effectively blocking the S-phase. This action is achieved by upregulating Bax, downregulating Bcl-2, and activating caspase-9 and caspase-3. The resultant cascade of events leads to the loss of mitochondrial membrane potential and the induction of cell apoptosis (Sui et al., 2016). The combined effects of Ligustrum lucidum and Epimedium are marked by a decrease in α-SMA and Ki-67 protein expression, LC3-II/LC3-I ratio, and Bcl-2/Bax protein ratio. Conversely, they result in an upregulation of caspase-3, p-mTOR, and Bax, all of which inhibit lung tissue cell proliferation and autophagy while promoting apoptosis within the lung tissue and BALF (Ma et al., 2020). Research by Huang et al. (Huang et al., 2022) emphasizes the potential of Hedyotis diffusa extract to regulate VDAC2/3 activity by promoting Bax and inhibiting Bcl-2. This dynamic interaction culminates in iron-induced apoptosis within lung adenocarcinoma cells. Poria cocos extract exerts cytotoxicity on human lung adenocarcinoma cells by elevating Bax expression and Bcl-2 phosphorylation levels. Additionally, it activates caspase-3 and induces apoptosis, an effect associated with mitochondrial perturbation (Lee et al., 2018). Moreover, Tetramethylpyrazine (TMP), as demonstrated by Jiang et al. (Jiang R. et al., 2021), diminishes the expression of NLRP3, curbing the formation of inflammatory complexes, thereby reducing caspase-1 activation, cell apoptosis, and inflammatory responses. Cinnamomum cassia extracts have a dose-dependent inhibition on NSCLC cell proliferation, both *in vivo* and *in vitro*. They also induce cell apoptosis (Wu et al., 2017). Polygonatum odoratum extract modulates miR-RNA, Akt-NF-κB, and Akt-mTOR, thereby stimulating apoptosis and autophagy in lung cancer (Wu et al., 2016). Polygonatum odoratum lectin was found to inhibit the Akt-NF-κB pathway to trigger cell apoptosis, simultaneously inducing autophagy by impeding the Akt-mTOR pathway (Li et al., 2014). Acetone extract of Bupleurum scorzonerifolium (AE-BS) exhibits a dose-dependent inhibitory impact on the proliferation of A549 human lung cancer cells. This is achieved through the inhibition of telomerase activity and the activation of cell apoptosis (Cheng et al., 2003). Pure compound dihydroisotanshinone I (DT) extracted from Salvia miltiorrhiza activates caspase-3/9 and PARP1, culminating in cell arrest in the G2/M phase (Wu et al., 2021). The mechanisms underlying traditional Chinese medicine-induced apoptosis in lung cancer cells predominantly involve the regulation of apoptosis-related genes (e.g., p53, C-myc, Bax, Bcl-2), modulation of intracellular Ca^2+^ concentrations and ACMP, activation of the caspase protease family, inhibition of telomerase activity, and other facets that ultimately induce cell apoptosis and achieve the desired therapeutic effect in NSCLC.

#### 2.1.3 Tumor angiogenesis

Tumor angiogenesis forms the cornerstone of tumor cell growth and metastasis, and the strategy for anti-tumor angiogenesis revolves around intervening in the regulatory factors of tumor vasculature and their functional connections. Among these factors, Vascular Endothelial Growth Factor (VEGF) and Cyclooxygenase-2 (COX-2) take center stage as pivotal vascular growth factors. Traditional Chinese medicine extracts have demonstrated their capability to hinder tumor growth and metastasis by effectively suppressing key vascular growth factors like VEGF and COX-2. For instance, the extract derived from Hedyotis diffusa exerts control over the Epithelial-Mesenchymal Transition (EMT) by inhibiting COX-2 protein expression. This action effectively curtails the metastatic potential of lung adenocarcinoma cells (Lin et al., 2019). In a separate study, Xie et al. (Xie et al., 2019) showcased the potential of DQA-modified paclitaxel plus ligustrazine micelles in downregulating the expression of VEGF, MMP-2, TGF-β1, and E-cadherin in lung cancer cells, thereby mitigating the metastatic tendencies of these cells. Furthermore, the extract of Ecliptae Herba has been found to significantly lower the levels of COX-2, TGF-β1, MMP-2, and α-SMA, while increasing the MMP-9/TIMP-1 ratio (You et al., 2015). This dual action serves to inhibit the formation of tumor blood vessels. Additionally, studies have revealed that the combination of Astragalus membranaceus and Angelica sinensis can reduce the expression of NF-κB, STAT3, HIF-1α, and VEGF, effectively impeding tumor growth in mice (Wu et al., 2019). Therefore, various traditional Chinese medicine monomers and extracts hold the potential to thwart tumor metastasis by intervening in tumor cell angiogenesis.

#### 2.1.4 Tumor microenvironment

Malignant tumors encompass a complex interplay between tumor cells and non-tumor cell components, including fibroblasts, endothelial cells, lymphocytes, macrophages, and extracellular matrix. This ensemble is collectively referred to as the tumor microenvironment. The tumor microenvironment plays a pivotal role in the initiation and progression of tumors, and contemporary research endeavors are dedicated to unraveling the intricate mechanisms through which the tumor microenvironment regulates tumor development. In recent years, investigations have underscored the capacity of traditional Chinese medicine extracts to exert anti-tumor effects by influencing the tumor microenvironment.

##### 2.1.4.1 Cell adhesion movement

Cell adhesion encompasses both homophilic adhesion between cancer cells and heterophilic adhesion between cancer cells and stromal cells. A reduction in the expression of specific adhesion molecules, such as E-cadherin, in tumor cells can lead to diminished homophilic cell adhesion. This, in turn, facilitates the detachment of tumor cells from the extracellular matrix. Epithelial-mesenchymal transition (EMT) denotes the transformation of epithelial cells into mesenchymal cells. It promotes cell metastasis and invasion, imbuing cells with stem cell characteristics, diminishing apoptosis and senescence, fostering immune evasion, and participating in physiological processes like tissue healing, organ fibrosis, and cancer initiation. Notably, during the EMT process, tumor cells develop resistance to chemotherapy and immunotherapy, allowing them to elude immune system surveillance. Metastasis stands as the primary factor contributing to the failure of lung cancer treatment, and inhibiting cell adhesion has emerged as a key focus of research within the realm of traditional Chinese medicine for curbing lung cancer metastasis. Xie et al. (Xie et al., 2019)observed that DQA-modified paclitaxel combined with ligustrazine micelles could downregulate the expression of VEGF, MMP2, TGF-β1, and E-cadherin in lung cancer cells, thus impeding lung cancer cell metastasis. Furthermore, research has revealed that the combination of Trichosanthin (TCS) and TRAIL can regulate the expression of apoptosis-related proteins (caspase 3/8 and PARP), endogenous apoptosis-related proteins (Bcl-2 and Bax), invasion-related proteins (E-cadherin, N-cadherin, Vimentin, ICAM-1, MMP-2, MMP-9), and cell cycle-related proteins (P27, CCNE1, and CDK2) (You et al., 2018). This orchestrated modulation leads to cell apoptosis and S-phase arrest, thereby suppressing cancer cell proliferation and invasion. Cinnamomum cassia extract (CCE) inhibits TGF-β1-induced motility and invasiveness of lung cancer cells by curtailing the adhesion of damaged cells to collagen (Lin et al., 2017). In the context of TGF-β1-induced EMT, dioscin, extracted from Rhizoma Dioscoreae Nipponicae, significantly heightens the expression of epithelial markers (E-cadherin) and reduces interstitial markers (N-cadherin and Snail). This serves to inhibit EMT, effectively restraining the *in vitro* migration and invasion of lung cancer cells (Lim et al., 2017). Paeonol inhibits TGF-β1-induced EMT while concurrently upregulating the expression of miR-126-5p in lung cancer cells. This dual action results in decreased cell viability, thereby inhibiting the survival and metastasis of human lung cancer cells (Lv et al., 2022).

##### 2.1.4.2 Extracellular matrix degradation

The successful invasion and metastasis of tumor cells within the body hinge on their ability to breach the formidable barriers presented by the basement membrane (BM) and the extracellular matrix (ECM), thereby gaining entry into the circulatory system. The pivotal steps in the invasion and metastasis process involve tumor cells initially adhering to extracellular matrix components (such as laminin, fibronectin, and collagen) through membrane surface receptors. Subsequently, they execute the degradation of the basement membrane and matrix by secreting an array of proteolytic enzymes. Finally, they undergo directed migration through the compromised basement membrane and matrix, ultimately entering the circulatory system. The expression and activity of matrix metalloproteinases (MMPs) are intricately linked with tumor invasion and metastasis, influencing various phases of tumor development. It is now established that traditional Chinese medicine can modulate the balance between matrix metalloproteinases (MMPs) and their inhibitors (TIMPs) in lung cancer tissue. This modulation leads to the inhibition of urokinase-type plasminogen activator (u-PA) and cathepsin B (CB) secretion, thereby preventing the degradation of the extracellular matrix by lung cancer cells. Dioscin, extracted from Rhizoma Dioscoreae Nipponicae, serves as a potent example of this action. It significantly diminishes the expression of p-AKT, MMP-2, and PCNA, effectively restraining the proliferation, invasion, and migration of A549 and PC-9 cells. This effect extends to a reduction in lung nodules, lung injury, and mortality in mouse lung cancer models (Xi et al., 2022). By inhibiting MMP-2 and u-PA, Cinnamomum cassia extract suppresses the extracellular matrix degradation by lung cancer cells, effectively mitigating TGF-β1-induced invasion and metastasis of lung cancer cells (Lin et al., 2017). Additionally, research by Gao et al. (Gao et al., 2012)underscores the potential of Angelica sinensis extract to reduce MMP-2, MMP-9, TGF-β1, and TIMP-1 expression while elevating TIMP-2 levels. This dual action inhibits the growth and metastasis of lung cancer cells. Sinomenine, extracted from Sinomenium acutum, is another promising compound that reduces the expression of MMP-2, MMP-9, and extracellular matrix metalloproteinase inducer (EMMPRIN/CD147), while concurrently increasing the expression of RECK, TIMP-1, and TIMP-2. This effectively impedes the migration and invasion of lung adenocarcinoma cells (Shen et al., 2020). Shen et al. (Shen et al., 2017) have also identified a similar anti-metastatic mechanism within the extract of Solanum nigrum. Furthermore, solasodine exhibits an ability to reduce the expression of MMP-2, MMP-9, and extracellular matrix metalloproteinase inducer (EMMPRIN) while elevating the expression of RECK, TIMP-1, and TIMP-2, thereby thwarting cell invasion. Through the utilization of gelatin zymography, which measures the activity of matrix metalloproteinases (MMP-2/-9) in NCI-H460 cells, it has been demonstrated that cantharidin inhibits MMP-2/-9 enzyme activity in NCI-H460 cells (Hsia et al., 2016). Hence, an array of traditional Chinese medicines and their extracts wield the potential to influence the trajectory of lung cancer through their impact on the tumor microenvironment.

#### 2.1.5 Body immunity function

Presently, research into the anti-tumor immune properties of active compounds within traditional Chinese medicine predominantly encompasses investigations into changes in immune cell activity, the expression of immune molecules, and the presentation of tumor antigens. Licorice extract, for instance, has demonstrated the ability to hinder the growth of NSCLC. It achieves this by elevating the abundance of PD-L1 protein, augmenting antigen presentation, and fostering the infiltration of CD8^+^ T cells (Zhu et al., 2021). Jiang et al. (Jiang R. et al., 2021)discovered that Tetramethylpyrazine (TMP) can inhibit the polarization of M1-type macrophages *in vitro*, while to some extent promoting the repolarization of M2-type macrophages. This effect results in the reduction of the transcription and secretion of inflammatory factors (IL-1β, IL-18, TNF-β) induced by lipopolysaccharide (LPS). In the context of lung cancer patients, the plasma activated by phytohemagglutination (PHA) exerts an inhibitory influence on lymphocyte proliferation, CD69 expression, and the production of perforin and granzyme B. This inhibition can be partially or completely reversed by Ganoderma lucidum polysaccharides (Gl-PS) (Sun et al., 2014). Astragalus polysaccharides (PG2) play a pivotal role in significantly augmenting the M1/M2 macrophage polarization rate of NSCLC cells. This action is coupled with a substantial inhibition of cell proliferation, clone formation, and tumor sphere formation. PG2 also promotes the functional maturation of dendritic cells, thus enhancing the T cell-mediated anti-cancer immune response (Bamodu et al., 2019). In an investigation focused on the impact of Trichosanthin (TCS) on the anti-tumor immune response in a 3LL Lewis lung cancer tumor model, the results revealed that TCS elevates the percentage of effector T cells. It fosters robust proliferation of antigen-specific effector T cells and significantly augments the secretion of Th1 cytokines. Moreover, it induces the generation of more memory T cells in tumor-bearing mice. This is accomplished through TCS upregulating the expression of the lung cancer 1 tumor suppressor factor (TSLC1) in 3LL tumor cells and elevating the expression of its ligand class I restrictive T cell-related molecule (CRTAM) in effector T cells. This enhancement, in turn, intensifies the anti-tumor response and induces immune protection (Cai et al., 2011). Furthermore, in their experiment, Zhang et al. (Zhang et al., 2015) noted that Atractylenolide I (AO-1) significantly curbed the production of TNF-α, IL-6, IL-1β, IL-13, and MIF in Bronchoalveolar Lavage Fluid (BALF), along with the production of BALF neutrophils and macrophages. Conversely, AO-1 was found to upregulate the production of IL-10 in BALF. Additionally, AO-I inhibited the expression of Toll-Like Receptor 4 (TLR4) and the activation of Nuclear Factor-Kappa B (NF-κB) induced by lipopolysaccharide (LPS).

#### 2.1.6 Signal transduction pathways

The regulation of cellular signaling pathways has emerged as a promising frontier in the battle against lung cancer. Tanshinone IIA, for instance, induces cell cycle arrest in the G1/S phase by orchestrating the CCNA2-CDK2 complex and the AURKA/PLK1 pathway, thereby promoting apoptosis in lung adenocarcinoma cells (Li et al., 2021). In a study by Lin et al. (Lin et al., 2022), Xanthotoxol extracted from Angelica dahurica demonstrated its capacity to trigger cell cycle arrest, foster cell apoptosis, and inhibit Epithelial-Mesenchymal Transition (EMT) processes by downregulating PI3K-AKT signaling. This dual action effectively suppresses the proliferation and metastasis of NSCLC. The administration of Ligustrazine yields significant reductions in the levels of Wnt and β-catenin. Additionally, it downregulates PTEN levels and interrupts the Wnt/β-catenin signaling pathway, resulting in the inhibition of lung cancer cell invasion and proliferation (Dong et al., 2020). Cantharidin exhibits the capability to inhibit the migration and invasion of lung cancer cells, a phenomenon closely associated with the activation of the PI3K-AKT signaling pathway (Kim et al., 2013). Ophiopogon japonicus extract drives an increase in the expression of autophagy-related mediators (including the LC3-II/LC3-I ratio, Atg-3, Atg-7, and Beclin-1) in A549 cells. This activation of autophagy in lung cancer cells is rooted in the inhibition of the PI3K/Akt/mTOR signaling pathway (Chen et al., 2017). Ligustilide, extracted from Angelica sinensis, effectively regulates the proliferation, apoptosis, and aerobic glycolysis of NSCLC cells through the PTEN/AKT signaling pathway (Jiang X. et al., 2021). Astragalus polysaccharides (APS) exert their anti-tumor effects by inhibiting the protein and gene expression of S1PR1, STAT3, and p-STAT3 in the S1PR1/STAT3 signaling pathway (Shen et al., 2023). Glycyrrhizin hampers the migration and invasion of lung cancer cells, an effect intricately linked to its reduction in the activity of the JAK/STAT signaling pathway (Wu et al., 2018). A study by Wang et al. (Wang et al., 2022)unveiled that catalpol, extracted from Radix Rehmanniae, hinders the Nrf2/ARE signaling pathway, leading to a substantial decrease in the proliferation and migration abilities of lung cancer cells. The primary mechanism behind the anti-lung cancer effect of Fritillaria extract revolves around protein phosphorylation. The extract successfully inhibits tumor cell proliferation and AKT phosphorylation, with a predominant role played by the PI3K/AKT signaling pathway (Zhao et al., 2023). Salvia chinensis extract effectively inhibits the transcriptional activity of the Wnt/β-catenin pathway, eliciting significant inhibitory effects on tumor cells *in vitro* (Wang et al., 2017). Meanwhile, glycyrrhizic acid regulates autophagy related to the PI3K/AKT/mTOR pathway, thereby inhibiting the production of inflammatory factors induced by lipopolysaccharide (LPS) in Acute Lung Injury (ALI) (Qu et al., 2019). A comprehensive summary of the specific extracts and their regulatory mechanisms within traditional Chinese medicine is provided in Table 1.

**TABLE 1:** Mechanism of action of traditional Chinese medicine extract in the treatment of non-small cell lung cancer.

Function mechanism		Traditional Chinese medicine and its extracts	Key gene expression	References
Cell proliferation		Licorice extract	downregulating the expression of CDK4-Cyclin complex	Zhu et al. (2021)
		Venenum Bufonis (resibufogenin)	downregulating cyclin D1 expression	Ichikawa et al. (2015)
		Polygonatum odoratum (Homoisoflavanone-1)	activating the p38/p53 signaling pathway	Ning et al. (2018)
		Salvia miltiorrhiza (tanshinone)	increasing the expression of p53 and p21, activating caspase-3/9 and PARP1	Ye et al. (2017)
Cell apoptosis		Angelica dahurica (imperatorin)	upregulating the gene expression of p53 and Bax, downregulating the expression of Mcl-1	Choochuay et al. (2013)
		Ethyl acetate extract from Selaginella doederleinii Hieron	upregulating Bax, downregulating Bcl-2, activating caspase-9 and caspase-3	Sui et al. (2016)
		The extract of Ligustrum lucidum and Epimedium	downregulating Bcl-2/Bax protein ratio, Bcl-2 and Beclin-1 protein and mRNA expression, upregulating caspase-3, p-mTOR and Bax	Ma et al. (2020)
		Acetone extract of Bupleurum scorzonerifolium	inhibiting telomerase activity	Cheng et al. (2003)
Tumor angiogenesis		Hedyotis diffusa (oldenlandia polysaccharide)	inhibiting COX-2 protein expression	Lin et al. (2019)
		Ligusticum wallichii (ligustrazine)	downregulating the expression of VEGF, MMP2, TGF-β1 and E-cadherin	Xie et al. (2019)
		Ecliptae Herba extract	reducing the levels of COX-2, TGF- β1, MMP-2 and α- SMA, increasing the MMP-9/TIMP-1 ratio	You et al. (2015)
		The extract of Astragalus membranaceus and Angelica sinensis	reducing the expression of NF-κβ, STAT3, HIF-1α and VEGF	Wu et al. (2019)
Tumor microenvironment	Cell adhesion movement	Ligusticum wallichii (Ligustrazine)	downregulating the expression of VEGF, MMP-2, TGF-β1 and E-cadherin	Xie et al. (2019)
		Trichosanthes kirilowi (Trichosanthin)	regulating the expression of E-cadherin, N-cadherin, Vimentin, ICAM-1, MMP-2 and MMP-9	You et al. (2018)
		Cinnamomum cassia extract	inhibiting the adhesion of damaged cells to collagen	Lin et al. (2017)
		Rhizoma Dioscoreae Nipponicae (dioscin)	increasing the expression of epithelial marker (E-cadherin) and interstitial marker (N-cadherin and Snail)	Lim et al. (2017)
	Extracellular matrix degradation	Cinnamomum cassia extract	inhibiting the expression of MMP-2 and uPA	Lin et al. (2017)
		Angelica sinensis extract	reducing MMP-2, MMP-9, TGF-β1 and TIMP-1, increasing TIMP-2	Gao et al. (2012)
		Sinomenium acutum (Sinomenine)	reducing the expression of MMP-2, MMP-9 and EMMPRIN, increasing the expression of RECK, TIMP-1 and TIMP-2	Shen et al. (2020)
		Solanum nigrum (Solasodine)		Shen et al. (2017)
Body immunity function		Licorice extract	increasing the abundance of PD-L1 protein, antigen presentation and infiltration of CD8+T cells	Zhu et al. (2021)
		Ligusticum wallichii (ligustrazine)	reducing the transcription and secretion of inflammatory factors (IL-1β, IL-18, TNF-β)	Jiang et al. (2021a)
		Astragalus membranaceus (Astragalus polysaccharides)	increasing the M1/M2 macrophage polarization rate of NSCLC cells	Bamodu et al. (2019)
		Trichosanthes kirilowi (Trichosanthin)	upregulating the expression of TSLC1 in 3LL tumor cells and CRTAM in effector T cells	Cai et al. (2011)
Signal transduction pathways		Ligusticum wallichii (Ligustrazine)	blocking the Wnt/β-catenin signaling pathway	Dong et al. (2020)
		Ophiopogon japonicus extract	inhibiting the PI3K/Akt/mTOR signaling pathway	Chen et al. (2017)
		Licorice (Glycyrrhizin)	inhibiting the JAK/STAT signaling pathway	Wu et al. (2018)
		Radix Rehmanniae (catalpol)	inhibiting the Nrf2/ARE signaling pathway	Wang et al. (2022)

### 2.2 Application of traditional Chinese medicine extract for non-small cell lung cancer

#### 2.2.1 Effect on chemotherapy drugs

Cisplatin (DDP) is a widely employed chemotherapy drug for lung cancer treatment (Ju et al., 2020; Liu et al., 2022). However, the development of gefitinib resistance (GR) is an inevitable challenge in the course of NSCLC therapy. Emerging research has shown that combining various traditional Chinese medicines with cisplatin can mitigate tumor cell resistance to cisplatin and enhance their anti-tumor potency. Bamodu et al. (Bamodu et al., 2019) reported a significant time-dependent reduction in the M2-associated tumor cell population with astragalus polysaccharides (PG2) treatment. This synergistically improved the anti-cancer efficacy of cisplatin on M2 cells and suppressed the growth of xenograft tumors in a NSCLC mouse model. Furthermore, the presence of PG2 significantly alleviated the side effects linked to cisplatin treatment. Cheng et al. (Cheng Z. et al., 2022)found that the extract of Ophiopogon japonicus exhibited a more pronounced cytotoxic effect on cisplatin-resistant lung cancer cells compared to normal tumor cells. Ophiopogonin B (OP-B) was shown to reduce tumor cell resistance to cisplatin by promoting Caspase-1/GSDMD-dependent pyroptosis. Ganoderma lucidum Polysaccharide (WSG), a glucose-rich polysaccharide derived from Ganoderma lucidum, demonstrated enhanced inhibition of lung cancer both *in vitro* and *in vivo* when combined with cisplatin. This combined treatment led to a synergistic reduction in the growth of lung cancer cells, enhancing the apoptosis response mediated by cisplatin. Additionally, WSG reduced the cytotoxic effects of cisplatin on macrophages and normal lung fibroblasts (Qiu et al., 2021). Artemisia argyi extract effectively suppressed both parent and gemcitabine-resistant lung cancer cells by inducing ROS production, mitochondrial membrane depolarization, and apoptosis, while mitigating Epithelial-Mesenchymal Transition (EMT) (Su et al., 2022). The ethanol extract of Centipeda minima intensified cisplatin-induced cell apoptosis in NSCLC xenografts (Gao et al., 2023). The anti-cancer efficacy of cordycepin, both *in vitro* and *in vivo*, was found to be comparable to afatinib and even superior to gefitinib. This suggests that cordycepin, either in isolation or combined with existing targeted therapies, might provide additional treatment options for drug-resistant NSCLC (Wei et al., 2019). The combination of Cordyceps sinensis extract Cordycepin and cisplatin exhibited a synergistic effect in inhibiting proliferation and promoting apoptosis in NSCLC cells, both in the presence and absence of cisplatin resistance. This anti-tumor activity was associated with the activation of AMPK and the inhibition of the AKT pathway, thereby enhancing the inhibitory effects of cisplatin on NSCLC (Liao et al., 2021). β-elemene, extracted from Radix curcuma wenyujin, acted synergistically with cisplatin against NSCLC cells by promoting apoptosis and impairing glucose metabolism, combating multidrug resistance, and preserving stemness (Cheng G. et al., 2022). The combination of lithospermum extract shikonin with gefitinib displayed a synergistic anti-tumor effect *in vitro* and *in vivo*. The potential molecular mechanisms appeared to be linked to the inhibition of PKM2/STAT3/cyclinD1 (Tang et al., 2018). Experiments have also revealed that gambogenic acid, an extract from garcinia, functioned as an inhibitor of the FGFR signaling pathway, thereby enhancing the efficacy of erlotinib when used in combination (Xu et al., 2018). Aucklandia lappa DC. extract significantly enhanced the effectiveness of gefitinib, suppressing the Muv phenotype of jgIs25. Meanwhile, it also downregulated the mRNA and protein expression of EGFR in jgIs25 (Huang et al., 2017). In a study by Lee et al. (Lee et al., 2011), a low dose of gefitinib, when combined with curcumin, exhibited a significant synergistic effect on apoptosis in a lung cancer cell line. This effect was achieved by reducing the mitochondrial membrane potential and activating caspase-9/caspase-3. Pristimerin inhibited cell proliferation, induced G0/G1 arrest, and promoted cell apoptosis in A549 and NCI-H446 cells. Pristimerin effectively synergized with cisplatin, resulting in the suppression of tumor growth (Zhang et al., 2019). In conclusion, traditional Chinese medicine extracts can synergistically enhance the anti-tumor effects of chemotherapy drugs.

#### 2.2.2 Effect on multidrug resistance

Multidrug resistance (MDR) in lung cancer cells remains a major obstacle to achieving satisfactory chemotherapy outcomes. Consequently, overcoming MDR in tumor cells to enhance chemotherapy effectiveness represents a crucial area of research. Trichosanthin (TCS), by upregulating DR4 and DR5, can trigger cell apoptosis, modulate invasion, influence cell cycle-related proteins, and heighten the sensitivity of NSCLC cells to TRAIL (You et al., 2018). Xu et al. (Xu et al., 2018) reported the successful use of gambogenic acid to overcome erlotinib resistance in NSCLC treatment through the inhibition of the FGFR signaling pathway when administered in combination. Andrographis paniculata addresses cisplatin resistance in tumor cells by acting on the miR-155-5p/SIRT1 axis (Pang et al., 2023). A synergistic inhibitory effect on lung cancer cell proliferation and invasion was observed with the combined use of Scutellaria baicalensis extract baicalin and cisplatin at specific doses and incubation times. The attenuation of cisplatin resistance was associated with the downregulation of MARK2 and p-Akt (Xu et al., 2017). Berberine, derived from Coptis chinensis extract, demonstrated its role as a naturally occurring MET inhibitor. It synergized with osimertinib in overcoming acquired resistance caused by MET amplification (Chen et al., 2022). Ming et al. (Ming et al., 2021) found that fucoxanthin, extracted from Laminaria Japonica, effectively inhibited tumor growth and heightened the sensitivity of lung cancer cells to gefitinib. This effect might be attributed to the inhibition of tumor cell proliferation and the activation of apoptosis. Artemisia annua extract dihydroartemisinin proved effective in overcoming acquired resistance to gefitinib in EGFR-mutant lung adenocarcinoma due to its STAT3 inhibitory activity and low toxicity (Xueting et al., 2017)^.^ Triptolide sensitized cancer cells to antitumor drugs both *in vitro* and in a xenograft tumor model system using lung carcinoma cells. This suggests that triptolide mitigates chemoresistance in cancer cells by targeting the Nrf2 pathway (Zhu et al., 2018). An extraction from Scutellariabarbata D. Don sensitized NSCLC cells to cisplatin treatment by downregulating the SHH signaling pathway (Chen WW. et al., 2021). Tripterygium wilfordii extract celastrol exhibited similar cytotoxic effects on both A549 parental and A549/DDP cisplatin-resistant cells. Celastrol also induced cell apoptosis in a dose- and time-dependent manner in both cell lines, accompanied by ROS accumulation, loss of mitochondrial membrane potentials, and cleavage of PARP and caspases (Shi, 2013). In summary, various traditional Chinese medicines effectively reverse the multidrug resistance observed in response to chemotherapeutic drugs. Their combined use enhances the sensitivity of these enhancers and significantly improves the effectiveness of tumor treatment. The applications of traditional Chinese medicine extracts for NSCLC are detailed in Table 2.

**TABLE 2:** Application of traditional Chinese medicine extract for non-small cell lung cancer.

Functional route	Traditional Chinese medicine and its extracts	Key gene expression	References
Effect on chemotherapy drugs	Ophiopogon japonicus (Ophiopogonin B)	reduces tumor cell resistance to cisplatin through the Caspase-1/GSDMD dependent pyroptosis pathway	Cheng et al. (2022a)
	Artemisia argyi extract	inducing ROS, mitochondrial membrane depolarization and apoptosis, reducing EMT	Su et al. (2022)
	Cordyceps sinensis (Cordycepin)	activating AMPK, inhibiting the AKT signaling pathway	Liao et al. (2021)
	Lithospermum (shikonin)	inhibiting PKM2/STAT3/cyclinD1	Tang et al. (2018)
	Garcinia (Gambogenic acid)	inhibiting the FGFR signaling pathway	Xu et al. (2018)
Multidrug resistance	Trichosanthes kirilowi (Trichosanthin)	upregulating DR4 and DR5	You et al. (2018)
	Scutellaria baicalensis (Baicalin)	downregulating MARK2 and p-Akt	Xu et al. (2017)
	Artemisia annua (Dihydroartemisinin)	inhibiting the STAT3 signaling pathway	Xueting et al. (2017)
	Scutellariabarbata D. Don extraction	downregulating the SHH signaling pathway	Chen et al. (2021a)
	Tripterygium wilfordii (Celastrol)	accumulating ROS, losing mitochondrial membrane potentials, slicing the dose-and-time dependence of PARP and caspases	Shi (2013)

### 2.3 The application of traditional Chinese medicine extract treatment for small cell lung cancer

Small cell lung cancer (SCLC) is an aggressive and recalcitrant malady, characterized by rapid growth and early metastatic spread to regional lymph nodes and distant sites. Chemotherapy is the general treatment for SCLC, however, majority of patients who typically exhibit a sensitive response to chemotherapy will swiftly relapse with relatively drug-resistant diseases (Waqar and Morgensztern, 2017; Mi et al., 2020). Despite considerably significant progress in targeted therapy and immunotherapy has been made in treating NSCLC, the therapies have not produced similar effects in SCLC (Byers and Rudin, 2015; Parikh et al., 2016). Although Traditional Chinese Medicine (TCM) is widely applied for patients with SCLC in China, the evidence of TCM in the treatment of SCLC is limited. Compared with chemotherapy alone, those with Chinese herbal medicine and chemotherapy had better therapeutic effects, including better KPS scores, and 5-year survival rate. Moreover, the incidence of gastrointestinal reaction and bone marrow depression was lower (Chen et al., 2020). TCM combined with chemotherapy may improve therapeutic effect, quality of life, and prolong survival time (Chen et al., 2020; Chen Y. et al., 2021; Xu et al., 2021; Qi et al., 2023). Aqueous extract of Artemisia annua played a preventive role against malignancy in the lung cancer models. It also induced apoptosis and decreased intracellular reactive oxygen species (ROS) in the lung cells (Allemailem, 2022). Peng et al. (Peng et al., 2021) revealed that the combination of anlotinib and Brucea Javanica Oil (BJO) significantly inhibited the growth of SCLC liver metastases and angiogenesis more than anlotinib monotherapy. In addition, BJO alleviated some of the side effects associated with anlotinib therapy. The results of the study indicated that the combination of anlotinib with BJO is promisingly active against liver metastases of SCLC, and has clinical potential. Moreover, the combination of gemcitabine (GEM) and BJO resulted in a reduced tumor growth rate and greater apoptosis compared to the vehicle control and GEM monotherapy (Yang et al., 2020). Therefore, traditional Chinese medicine extracts have potential clinical application in SCLC.

## 3 Treatment of other lung-related diseases with traditional Chinese medicine extracts

### 3.1 Pulmonary fibrosis

In recent years, the aging trend within China’s population has become increasingly prominent, and the incidence of chronic respiratory diseases has been steadily rising. Furthermore, pulmonary fibrosis, as a debilitating interstitial lung condition, presents limited treatment options. Ecliptae Herba extract has shown remarkable efficacy in ameliorating pathological lung tissue changes by reducing the levels of hydroxyproline (HYP), increasing superoxide dismutase (SOD) activity, and decreasing malondialdehyde (MDA) content. This extract, by mitigating oxidative stress, lung tissue inflammation, and epithelial-mesenchymal transition (EMT), exhibits a protective role against pulmonary fibrosis induced by bleomycin (You et al., 2015). Extracts from Radix Pseudostellariae, notably Heterophyllin B, have been found to significantly inhibit EMT and the redifferentiation of lung fibroblasts. Furthermore, it offers protection against bleomycin-induced pulmonary fibrosis by modulating the STING signaling mediated through AMPK and TGF-Smad2/3 pathways (Shi et al., 2022). Catalpol, extracted from Radix Rehmanniae, effectively inhibits NF-κB and MAPK signaling pathways mediated by TLR4, ultimately alleviating bleomycin-induced pulmonary fibrosis (Fu et al., 2014). Meanwhile, Icaritin plays a pivotal role in downregulating the expression of α-SMA and collagen I, effectively inhibiting myofibroblast differentiation, which suggests its potential in treating pulmonary fibrosis (Hua et al., 2021). Furthermore, the research by Li et al. (Li S. et al., 2022) demonstrates the inhibitory effects of pheretima protein on extracellular matrix (ECM), EMT, and anti-inflammatory markers, which contribute to the amelioration of bleomycin-induced pulmonary fibrosis. Alcohol extracts from Sceptridium ternatum (Thunb.) Lyon have exhibited anti-pulmonary fibrosis effects by targeting the SETDB1/STAT3/p-STAT3 signaling pathway (Zou et al., 2023). Qian et al. (Qian et al., 2018) have shown that Astragalus IV significantly inhibits FOXO3a hyperphosphorylation and downregulation induced by TGF-β1/PI3K/Akt, effectively reversing EMT during pulmonary fibrosis progression. Astragalus also exerts antifibrotic effects by inhibiting EMT, ROS, TGF-β1/Smads, apoptosis, and inflammatory pathways (Zhu et al., 2022). Euodia rutaecarpa (Juss.) Benth. extract evodiamine has demonstrated the ability to protect against pulmonary fibrosis-induced inflammatory damage both *in vitro* and *in vivo* by inhibiting apoptosis, inflammatory cytokine release, and activating the apelin pathway (Ye et al., 2021). Moreover, Snithin has significantly mitigated bleomycin-induced pulmonary fibrosis by downregulating the mechanism of TGF-β1/NOX4-mediated oxidative stress in lung fibroblasts (Fang et al., 2021). Salvia miltiorrhiza extract Tanshinone IIA has been instrumental in regulating the Keap1/Nrf2 signaling pathway through the activation of Sestrin2, effectively inhibiting pulmonary fibrosis (Li H. et al., 2022). Shenks exhibits its anti-fibrotic properties by blocking the TGF-β1 pathway and regulating oxidative/antioxidant balance (Chu et al., 2017). Peimine has contributed to the amelioration of pulmonary fibrosis by inhibiting M2-type macrophage polarization through the suppression of P38/Akt/STAT6 signals (Cai et al., 2022).

### 3.2 Acute lung injury

Acute lung injury, one of the most prevalent and severe respiratory conditions, currently lacks effective treatment options. Catalpol has proven to be effective in protecting against lipopolysaccharide-induced acute lung injury (Fu et al., 2014). Similarly, glycyrrhizic acid has shown promise in improving sepsis-induced acute lung injury (ALI)/acute respiratory distress syndrome (ARDS) by inhibiting HMGB1 and ameliorating inflammation in experimental sepsis rat models (Shen et al., 2022). Astragaloside IV, acting through the TLR4/NF-κB signaling pathway, has demonstrated its ability to induce cell autophagy, which, in turn, inhibits the release of inflammatory cytokines and improves the overall inflammatory response (Ying et al., 2021). Indigoin has offered relief in cases of lipopolysaccharide-induced acute lung injury by reducing malondialdehyde (MDA) levels and IL-1β and TNF-α expression in mice (Qi et al., 2017). Spatholobus stem extract has ameliorated acute lung inflammation induced by IAV by inhibiting the MAPK, PI3K-AKT, and oxidative stress pathways (Cai and Zhang, 2022). Furthermore, Euphorbia factor L2 (EFL2), derived from marijuana extract, has exhibited anti-inflammatory effects in a murine acute lung injury model by reducing the levels of inflammatory markers such as interleukin-1 β (IL-1β), interleukin-6 (IL-6), tumor necrosis factor-α (TNF-α), interleukin-8 (IL-8), and myeloperoxidase (MPO) in lung and bronchioalveolar lavage fluid (Zhang et al., 2018). Ren et al. (Ren et al., 2023) found that ergolactone, through its irreversible binding to the NACHT domain of NLRP3, effectively blocked the assembly and activation of NLRP3 inflammasomes. In a lipopolysaccharide-induced acute lung injury model, ergolactone alleviated acute lung injury in wild-type mice. Astragaloside IV targeted PRDX6 and inhibited the activation of the RAC subunit in NADPH oxidase 2, thus suppressing oxidative damage and inflammation in mice with acute lung injury (Cheng et al., 2023). Traditional Chinese medicines, along with their bioactive compounds, such as epigallocatechin-3-gallate (EGCG), kaempferol, isorhamnetin, quercetin, and β-sitosterol, predominantly regulate NF-κB, Nrf2/HO-1, NLRP3, TGF-β/Smad, MAPK, and PI3K/Akt/mTOR pathways, successfully mitigating infection and inflammation across a spectrum of lung-related diseases, including acute lung injury, chronic obstructive pulmonary disease, pulmonary fibrosis, asthma, and lung cancer (Wang et al., 2021). These findings underscore the pivotal role of traditional Chinese medicine extracts in the treatment of lung-related diseases, such as acute lung injury.

### 3.3 Other lung-related diseases

The incidence of various lung-related diseases, including cor pulmonale, chronic obstructive pulmonary disease, pulmonary tuberculosis, and asthma, has been steadily on the rise. It is imperative to acknowledge the clinical significance of these diseases. Traditional Chinese medicines have shown promise in addressing these conditions. Astragalus, radix paeoniae rubra, and Psoralea corylifolia have demonstrated their efficacy in reducing pulmonary keratinocytes growth factor (KGF), secretory immunoglobulin A (sIgA), and collagen fiber deposition. These effects lead to improved lung function and provide a valuable treatment approach for cor pulmonale (Jing et al., 2017). Resveratrol, extracted from Polygonum cuspidatum, acts as an activator of SIRT1 and has the potential to alleviate chronic obstructive pulmonary disease by activating the SIRT1 pathway (Ma and Li, 2020). Anemoside B4 has shown significant promise in attenuating the increased levels of inflammatory markers, including IL-1β, TGF-β, IL-8, and TNF-α, in human bronchial epithelial cells exposed to cigarette smoke extract (CSE). This is achieved by inhibiting the MAPK/AP-1/TGF-β signaling pathway, thereby preventing chronic pulmonary obstructive disease induced by cigarette smoke (Ma et al., 2022). Isoliquiritigenin effectively reduces inflammation induced by *Mycobacterium tuberculosis* (Mtb) through the Notch1/NF-κB and MAPK signaling pathways (Sun et al., 2022). Icariin, characterized by its anti-inflammatory, antioxidant, and immune-regulating properties, presents a multi-target intervention approach to asthma treatment (Yuan et al., 2022). Safranal, an extract from saffron crocus, has been shown to alleviate OVA-induced asthma by inhibiting the MAPK and NF-κB pathways, thus inhibiting mast cell activation and the production of proinflammatory factors (Lertnimitphun et al., 2021). The specific extracts and regulatory mechanisms of traditional Chinese medicine are detailed in Table 3, underscoring the potential of these remedies in addressing a variety of lung-related diseases.

**TABLE 3:** Mechanism of action of traditional Chinese medicine extract in treating other lung-related diseases.

Diseases	Traditional Chinese medicine and its extracts	Key gene expression	References
Pulmonary fibrosis	Epimedium (Icaritin)	reducing the expression of α- SMA and collagen I	Hua et al. (2021)
	Earthworm (Pheretima protein)	inhibiting ECM, EMT, and anti-inflammatory markers	Li et al. (2022a)
	Astragalus membranaceus (Astragalus)	inhibiting EMT, ROS, TGF-β1/Smads	Zhu et al. (2022)
	Hedyotis diffusa (Shenks)	blocking TGF-β1 pathway and regulating oxidative/antioxidant balance	Chu et al. (2017)
Acute lung injury	Licorice (Glycyrrhizic acid)	improving ALI/ARDS and inflammation by inhibiting HMBG1	Shen et al. (2022)
	Spatholobus stem extract	inhibiting MAPK, PI3K-AKT, and oxidative stress pathways	Cai and Zhang (2022)
	Marijuana (Euphorbia factor L2)	reducing the levels of IL-1 β, IL-6, TNF-α, IL-8 and MPO	Zhang et al. (2018)
	Astragalus membranaceus (Astragaloside IV)	inhibiting activation of RAC subunit in NADPH oxidase 2	Cheng et al. (2023)
Other lung-related diseases	The extract of Astragalus, radix paeoniae rubra, and Psoralea corylifolia	reducing KGF, sIgA, and collagen fiber deposition	Jing et al. (2017)
	Polygonum cuspidatum (Resveratrol)	activating the SIRT1 pathway	Ma and Li (2020)
	Licorice (Isoliquiritigenin)	reducing the Notch1/NF-κB and MAPK signaling pathways	Sun et al. (2022)
	Saffron crocus (Safranal)	inhibiting the MAPK and NF-κB pathways	Lertnimitphun et al. (2021)

## 4 Discussion and prospect

In conclusion, traditional Chinese medicine offers a multifaceted and layered approach to the prevention and treatment of lung cancer. Experimental research on the anti-lung cancer effects of traditional Chinese medicine has progressed from morphological observations to the microscopic levels of cells, molecules, and genes. Numerous studies have shed light on the mechanisms through which traditional Chinese medicine inhibits lung cancer growth and metastasis. These mechanisms encompass inhibiting tumor cell proliferation, inducing tumor cell apoptosis, and suppressing tumor angiogenesis. Traditional Chinese medicine also affects the cellular microenvironment, regulates immune function, modulates signal transduction pathways, and reverses multidrug resistance in tumor cells. These findings provide a robust scientific foundation for lung cancer treatment with traditional Chinese medicine and contribute to the advancement of the traditional Chinese medicine industry.

However, several challenges persist, including:

1. Overemphasis on traditional Chinese medicine compound formulas and active ingredients at the expense of single Chinese medicine and extracts in anti-lung cancer research.
2. The complex, multifaceted mechanisms of action in traditional Chinese medicine extracts, which pose challenges in pharmacokinetic research, such as determining quantitative standards, sampling times, and concentration standards in plasma or serum.
3. Interferences and uncertainties associated with single drugs and their traditional Chinese medicine extracts, raising doubts about result accuracy.
4. A predominant focus on gene regulation research in the mechanism of action, with less exploration of the interplay between various gene regulations.
5. A predominance of *in vitro* studies with limited research on *in vivo* cell apoptosis, hindering the understanding of drug metabolism and induced cell apoptosis *in vivo*.

To address these issues, we propose that future research and development of anti-lung cancer effects of traditional Chinese medicine single drugs and their extracts should involve interdisciplinary collaboration. This approach, guided by traditional Chinese medicine theory, can delve into the complex biological processes underlying tumor initiation, development, and metastasis. The goal is to develop effective formulations that target lung cancer cells through multiple avenues, including various targets, pathways, and methods. Modern pharmacology, immunology, and molecular biology are steadily unraveling the anti-lung cancer mechanisms of traditional Chinese medicine. Continuous research and exploration in this field aim to harness the full clinical therapeutic potential of traditional Chinese medicine in lung cancer treatment.

## References

[B1] Allemailem K. S. (2022). Aqueous extract of Artemisia annua shows *in vitro* antimicrobial activity and an *in vivo* chemopreventive effect in a small-cell lung cancer model. Plants (Basel) 11 (23), 3341. 10.3390/plants11233341 36501380 PMC9739242

[B2] Bamodu O. A. Kuo K. T. Wang C. H. Huang W. C. Wu A. T. H. Tsai J. T. (2019). Astragalus polysaccharides (PG2) enhances the M1 polarization of macrophages, functional maturation of dendritic cells, and T cell-mediated anticancer immune responses in patients with lung cancer. Nutrients 11 (10), 2264. 10.3390/nu11102264 31547048 PMC6836209

[B3] Byers L. A. Rudin C. M. (2015). Small cell lung cancer: where do we go from here? Cancer 121 (5), 664–672. 10.1002/cncr.29098 25336398 PMC5497465

[B4] Cai W. Zhang S. L. (2022). Anti-inflammatory mechanisms of total flavonoids from mosla scabra against influenza A virus-induced pneumonia by integrating network pharmacology and experimental verification. Evid. Based Complement. Altern. Med. 2022, 2154485, 2154485. 10.1155/2022/2154485 PMC9200497 35722153

[B5] Cai Y. Xiong S. Zheng Y. Luo F. Jiang P. Chu Y. (2011). Trichosanthin enhances anti-tumor immune response in a murine Lewis lung cancer model by boosting the interaction between TSLC1 and CRTAM. Cell Mol. Immunol. 8 (4), 359–367. 10.1038/cmi.2011.12 21572449 PMC4002443

[B6] Cai Z. H. Tian Y. G. Li J. Z. Zhao P. Li J. S. Mei X. (2022). Peimine ameliorates pulmonary fibrosis via the inhibition of M2-type macrophage polarization through the suppression of P38/Akt/STAT6 signals. Biosci. Rep. 42 (10), BSR20220986. 10.1042/BSR20220986 36111628 PMC9547170

[B7] Chen J. Yuan J. Zhou L. Zhu M. Shi Z. Song J. (2017). Regulation of different components from Ophiopogon japonicus on autophagy in human lung adenocarcinoma A549Cells through PI3K/Akt/mTOR signaling pathway. Biomed. Pharmacother. 87, 118–126. 10.1016/j.biopha.2016.12.093 28049093

[B8] Chen S. Bao Y. Xu J. Zhang X. He S. Zhang Z. (2020). Efficacy and safety of TCM combined with chemotherapy for SCLC: a systematic review and meta-analysis. J. Cancer Res. Clin. Oncol. 146 (11), 2913–2935. 10.1007/s00432-020-03353-0 32797283 PMC11804371

[B9] Chen W. W. Gong K. K. Yang L. J. Dai J. J. Zhang Q. Wang F. (2021a). Scutellariabarbata D. Don extraction selectively targets stemness-prone NSCLC cells by attenuating SOX2/SMO/GLI1 network loop. J. Ethnopharmacol. 265, 113295. 10.1016/j.jep.2020.113295 32841701

[B10] Chen Y. Yu M. Liu Z. Zhang Y. Li Q. Yang G. (2021b). Effects of traditional Chinese medicine combined with chemotherapy for extensive-stage small-cell lung cancer patients on improving oncologic survival: study protocol of a multicenter, randomized, single-blind, placebo-controlled trial. Trials 22 (1), 437. 10.1186/s13063-021-05407-1 34238342 PMC8265049

[B11] Chen Z. Vallega K. A. Chen H. Zhou J. Ramalingam S. S. Sun S. Y. (2022). The natural product berberine synergizes with osimertinib preferentially against MET-amplified osimertinib-resistant lung cancer via direct MET inhibition. Pharmacol. Res. 175, 105998. 10.1016/j.phrs.2021.105998 34826601 PMC8755628

[B12] Cheng C. Liu K. Shen F. Zhang J. Xie Y. Li S. (2023). Astragaloside IV targets PRDX6, inhibits the activation of RAC subunit in NADPH oxidase 2 for oxidative damage. Phytomedicine 114, 154795. 10.1016/j.phymed.2023.154795 37030053

[B13] Cheng G. Li L. Li Q. Lian S. Chu H. Ding Y. (2022b). β-elemene suppresses tumor metabolism and stem cell-like properties of non-small cell lung cancer cells by regulating PI3K/AKT/mTOR signaling. Am. J. Cancer Res. 12 (4), 1535–1555. 35530288 PMC9077083

[B14] Cheng Y. L. Chang W. L. Lee S. C. Liu Y. G. Lin H. C. Chen C. J. (2003). Acetone extract of Bupleurum scorzonerifolium inhibits proliferation of A549 human lung cancer cells via inducing apoptosis and suppressing telomerase activity. Life Sci. 73 (18), 2383–2394. 10.1016/s0024-3205(03)00648-9 12941440

[B15] Cheng Z. Li Z. Gu L. Li L. Gao Q. Zhang X. (2022a). Ophiopogonin B alleviates cisplatin resistance of lung cancer cells by inducing Caspase-1/GSDMD dependent pyroptosis. J. Cancer 13 (2), 715–727. 10.7150/jca.66432 35069914 PMC8771518

[B16] Choochuay K. Chunhacha P. Pongrakhananon V. Luechapudiporn R. Chanvorachote P. (2013). Imperatorin sensitizes anoikis and inhibits anchorage-independent growth of lung cancer cells. J. Nat. Med. 67 (3), 599–606. 10.1007/s11418-012-0719-y 23108812

[B17] Chu H. Shi Y. Jiang S. Zhong Q. Zhao Y. Liu Q. (2017). Treatment effects of the traditional Chinese medicine Shenks in bleomycin-induced lung fibrosis through regulation of TGF-beta/Smad3 signaling and oxidative stress. Sci. Rep. 7 (1), 2252. 10.1038/s41598-017-02293-z 28533545 PMC5440393

[B18] Dong Y. Yang Y. Wei Y. Gao Y. Jiang W. Wang G. (2020). Ligustrazine eases lung cancer by regulating PTEN and Wnt/β-catenin pathway. Transl. Cancer Res. 9 (3), 1742–1751. 10.21037/tcr.2020.03.26 35117521 PMC8798425

[B19] Fang L. Wang W. Chen J. Zuo A. Gao H. Yan T. (2021). Osthole attenuates bleomycin-induced pulmonary fibrosis by modulating NADPH oxidase 4-derived oxidative stress in mice. Oxid. Med. Cell Longev. 2021, 3309944, 3309944. 10.1155/2021/3309944 34527170 PMC8437590

[B20] Fu K. Piao T. Wang M. Zhang J. Jiang J. Wang X. (2014). Protective effect of catalpol on lipopolysaccharide-induced acute lung injury in mice. Int. Immunopharmacol. 23 (2), 400–406. 10.1016/j.intimp.2014.07.011 25063711

[B21] Gao C. Pan H. Ma F. Zhang Z. Zhao Z. Song J. (2023). Centipeda minima active components and mechanisms in lung cancer. BMC Complement. Med. Ther. 23 (1), 89. 10.1186/s12906-023-03915-y 36959600 PMC10035269

[B22] Gao M. Zhang J. H. Zhou F. X. Xie C. H. Han G. Fang S. Q. (2012). Angelica sinensis suppresses human lung adenocarcinoma A549 cell metastasis by regulating MMPs/TIMPs and TGF-β1. Oncol. Rep. 27 (2), 585–593. 10.3892/or.2011.1527 22076386

[B23] Goldstraw P. Crowley J. Chansky K. Giroux D. J. Groome P. A. Rami-Porta R. (2007). The IASLC Lung Cancer Staging Project: proposals for the revision of the TNM stage groupings in the forthcoming (seventh) edition of the TNM Classification of malignant tumours. J. Thorac. Oncol. 2 (8), 706–714. 10.1097/JTO.0b013e31812f3c1a 17762336

[B24] Hsia T. C. Yu C. C. Hsiao Y. T. Wu S. H. Bau D. T. Lu H. F. (2016). Cantharidin impairs cell migration and invasion of human lung cancer NCI-H460 cells via UPA and MAPK signaling pathways. Anticancer Res. 36 (11), 5989–5997. 10.21873/anticanres.11187 27793925

[B25] Hua Q. Huang X. Xie W. Zhao F. Cheng H. Luo Z. (2021). PPARγ mediates the anti-pulmonary fibrosis effect of icaritin. Toxicol. Lett. 350, 81–90. 10.1016/j.toxlet.2021.06.014 34153405

[B26] Huang F. Pang J. Xu L. Niu W. Zhang Y. Li S. (2022). Hedyotis diffusa injection induces ferroptosis via the Bax/Bcl2/VDAC2/3 axis in lung adenocarcinoma. Phytomedicine 104, 154319. 10.1016/j.phymed.2022.154319 35853302

[B27] Huang G. Tong Y. He Q. Wang J. Chen Z. (2017). Aucklandia lappa DC. extract enhances gefitinib efficacy in gefitinib-resistance secondary epidermal growth factor receptor mutations. J. Ethnopharmacol. 206, 353–362. 10.1016/j.jep.2017.06.011 28619365

[B28] Ichikawa M. Sowa Y. Iizumi Y. Aono Y. Sakai T. (2015). Resibufogenin induces G1-phase arrest through the proteasomal degradation of cyclin D1 in human malignant tumor cells. PLoS One 10 (6), e0129851. 10.1371/journal.pone.0129851 26121043 PMC4488249

[B29] Jiang R. Xu J. Zhang Y. Zhu X. Liu J. Tan Y. (2021a). Ligustrazine alleviate acute lung injury through suppressing pyroptosis and apoptosis of alveolar macrophages. Front. Pharmacol. 12, 680512. 10.3389/fphar.2021.680512 34122107 PMC8193053

[B30] Jiang X. Zhao W. Zhu F. Wu H. Ding X. Bai J. (2021b). Ligustilide inhibits the proliferation of non-small cell lung cancer via glycolytic metabolism. Toxicol. Appl. Pharmacol. 410, 115336. 10.1016/j.taap.2020.115336 33212065

[B31] Jing Y. Zhang H. Cai Z. Zhao Y. Wu Y. Zheng X. (2017). Bufei huoxue capsule attenuates pm2.5-induced pulmonary inflammation in mice. Evid. Based Complement. Altern. Med. 2017, 1575793. 10.1155/2017/1575793 PMC5350288 28337225

[B32] Ju Z. S. Sun B. Bao D. Zhang X. F. (2020). Effect of lncRNA-BLACAT1 on drug resistance of non-small cell lung cancer cells in DDP chemotherapy by regulating cyclin D1 expression. Eur. Rev. Med. Pharmacol. Sci. 24 (18), 9465–9472. 10.26355/eurrev_202009_23031 33015788

[B33] Kim Y. M. Ku M. J. Son Y. J. Yun J. M. Kim S. H. Lee S. Y. (2013). Anti-metastatic effect of cantharidin in A549 human lung cancer cells. Arch. Pharm. Res. 36 (4), 479–484. 10.1007/s12272-013-0044-3 23435912

[B34] Latrèche A. Bourbonne V. Lucia F. (2022). Unrecognized thoracic radiotherapy toxicity: a review of literature. Cancer Radiother. 26 (4), 616–621. 10.1016/j.canrad.2021.10.008 35153153

[B35] Lee J. Y. Lee Y. M. Chang G. C. Yu S. L. Hsieh W. Y. Chen J. J. (2011). Curcumin induces EGFR degradation in lung adenocarcinoma and modulates p38 activation in intestine: the versatile adjuvant for gefitinib therapy. PLoS One 6 (8), e23756. 10.1371/journal.pone.0023756 21858220 PMC3157465

[B36] Lee S. Lee S. Roh H. S. Song S. S. Ryoo R. Pang C. (2018). Cytotoxic constituents from the sclerotia of poria cocos against human lung adenocarcinoma cells by inducing mitochondrial apoptosis. Cells 7 (9), 116. 10.3390/cells7090116 30149516 PMC6162800

[B37] Lertnimitphun P. Zhang W. Fu W. Yang B. Zheng C. Yuan M. (2021). Safranal alleviated OVA-induced asthma model and inhibits mast cell activation. Front. Immunol. 12, 585595. 10.3389/fimmu.2021.585595 34093515 PMC8173045

[B38] Li C. Chen J. Lu B. Shi Z. Wang H. Zhang B. (2014). Molecular switch role of Akt in Polygonatum odoratum lectin-induced apoptosis and autophagy in human non-small cell lung cancer A549 cells. PLoS One 9 (7), e101526. 10.1371/journal.pone.0101526 24992302 PMC4081584

[B39] Li C. Lei S. Ding L. Xu Y. Wu X. Wang H. (2023). Global burden and trends of lung cancer incidence and mortality. Chin. Med. J. Engl. 136 (13), 1583–1590. 10.1097/CM9.0000000000002529 37027426 PMC10325747

[B40] Li H. Wu M. Guo C. Zhai R. Chen J. (2022b). Tanshinone IIA regulates keap1/nrf2 signal pathway by activating Sestrin2 to restrain pulmonary fibrosis. Am. J. Chin. Med. 50 (8), 2125–2151. 10.1142/S0192415X22500914 36309810

[B41] Li S. Yang Q. Chen F. Tian L. Huo J. Meng Y. (2022a). The antifibrotic effect of pheretima protein is mediated by the TGF-β1/Smad2/3 pathway and attenuates inflammation in bleomycin-induced idiopathic pulmonary fibrosis. J. Ethnopharmacol. 286, 114901, 114901. 10.1016/j.jep.2021.114901 34890730

[B42] Li Z. Zhang Y. Zhou Y. Wang F. Yin C. Ding L. (2021). Tanshinone IIA suppresses the progression of lung adenocarcinoma through regulating CCNA2-CDK2 complex and AURKA/PLK1 pathway. Sci. Rep. 11 (1), 23681. 10.1038/s41598-021-03166-2 34880385 PMC8654884

[B43] Liao X. Z. Gao Y. Zhao H. W. Zhou M. Chen D. L. Tao L. T. (2021). Cordycepin reverses cisplatin resistance in non-small cell lung cancer by activating AMPK and inhibiting AKT signaling pathway. Front. Cell Dev. Biol. 8, 609285. 10.3389/fcell.2020.609285 33520990 PMC7843937

[B44] Lim W. C. Kim H. Kim Y. J. Choi K. C. Lee I. H. Lee K. H. (2017). Dioscin suppresses TGF-β1-induced epithelial-mesenchymal transition and suppresses A549 lung cancer migration and invasion. Bioorg Med. Chem. Lett. 27 (15), 3342–3348. 10.1016/j.bmcl.2017.06.014 28610976

[B45] Lin C. Y. Hsieh Y. H. Yang S. F. Chu S. C. Chen P. N. Hsieh Y. S. (2017). Cinnamomum cassia extracts reverses TGF-β1-induced epithelial-mesenchymal transition in human lung adenocarcinoma cells and suppresses tumor growth *in vivo* . Environ. Toxicol. 32 (7), 1878–1887. 10.1002/tox.22410 28258635

[B46] Lin L. Cheng K. He Z. Lin Q. Huang Y. Chen C. (2019). A polysaccharide from Hedyotis diffusa interrupts metastatic potential of lung adenocarcinoma A549 cells by inhibiting EMT via EGFR/Akt/ERK signaling pathways. Int. J. Biol. Macromol. 129, 706–714. 10.1016/j.ijbiomac.2019.02.040 30738900

[B47] Lin X. Liu J. Zou Y. Tao C. Chen J. (2022). Xanthotoxol suppresses non-small cell lung cancer progression and might improve patients' prognosis. Phytomedicine 105, 154364. 10.1016/j.phymed.2022.154364 35932608

[B48] Liu J. H. Yang H. L. Deng S. T. Hu Z. Chen W. F. Yan W. W. (2022). The small molecule chemical compound cinobufotalin attenuates resistance to DDP by inducing ENKUR expression to suppress MYH9-mediated c-Myc deubiquitination in lung adenocarcinoma. Acta Pharmacol. Sin. 43 (10), 2687–2695. 10.1038/s41401-022-00890-x 35296779 PMC9525298

[B49] Lv J. Zhu S. Chen H. Xu Y. Su Q. Yu G. (2022). Paeonol inhibits human lung cancer cell viability and metastasis *in vitro* via miR-126-5p/ZEB2 axis. Drug Dev. Res. 83 (2), 432–446. 10.1002/ddr.21873 34636432

[B50] Lv Y. X. Pan H. R. Song X. Y. Chang Q. Q. Zhang D. D. (2021). Hedyotis diffusa plus Scutellaria barbata suppress the growth of non-small-cell lung cancer via NLRP3/NF-κB/MAPK signaling pathways. Evid. Based Complement. Altern. Med. 2021, 6666499, 6666499. 10.1155/2021/6666499 PMC8233093 34239588

[B51] Ma B. N. Li X. J. (2020). Resveratrol extracted from Chinese herbal medicines: a novel therapeutic strategy for lung diseases. Chin. Herb. Med. 12 (4), 349–358. 10.1016/j.chmed.2020.07.003 32963508 PMC7498443

[B52] Ma H. Zhou Z. Chen L. Wang L. Muge Q. (2022). Anemoside B4 prevents chronic obstructive pulmonary disease through alleviating cigarette smoke-induced inflammatory response and airway epithelial hyperplasia. Phytomedicine 107, 154431. 10.1016/j.phymed.2022.154431 36115169

[B53] Ma Z. Tang X. Gao Y. Wang H. Yu P. Liu R. (2020). Combined extracts of epimedii folium and ligustri lucidi fructus with budesonide attenuate airway remodeling in the asthmatic rats by regulating apoptosis and autophagy. Evid. Based Complement. Altern. Med. 2020, 2319409, 2319409. 10.1155/2020/2319409 PMC7426755 32831860

[B54] Mi X. Zhang X. He S. Zhang Z. Qi R. Jiang J. (2020). Chinese herbal medicine for small cell lung cancer patients: a protocol for a systematic review and meta-analysis. Med. Baltim. 99 (52), e23746. 10.1097/MD.0000000000023746 PMC7769348 33350758

[B55] Ming J. X. Wang Z. C. Huang Y. Ohishi H. Wu R. J. Shao Y. (2021). Fucoxanthin extracted from Laminaria Japonica inhibits metastasis and enhances the sensitivity of lung cancer to Gefitinib. J. Ethnopharmacol. 265, 113302. 10.1016/j.jep.2020.113302 32860893

[B56] Ning D. Jin M. Xv T. Sun J. Li M. (2018). Homoisoflavanone-1 isolated from Polygonatum odoratum arrests the cell cycle and induces apoptosis in A549 cells. Oncol. Lett. 16 (3), 3545–3554. 10.3892/ol.2018.9085 30127960 PMC6096101

[B57] Pang C. Zhang T. Chen Y. Yan B. Chen C. Zhang Z. (2023). Andrographis modulates cisplatin resistance in lung cancer via miR-155-5p/SIRT1 axis. Funct. Integr. Genomics 23 (3), 260. 10.1007/s10142-023-01186-x 37530871

[B58] Parikh M. Riess J. Lara P. N. Jr (2016). New and emerging developments in extensive-stage small cell lung cancer therapeutics. Curr. Opin. Oncol. 28 (2), 97–103. 10.1097/CCO.0000000000000264 26844984 PMC5139911

[B59] Peng S. Dong W. Chu Q. Meng J. Yang H. DU Y. (2021). Traditional Chinese medicine Brucea javanica Oil enhances the efficacy of anlotinib in a mouse model of liver-metastasis of small-cell lung cancer. Vivo 35 (3), 1437–1441. 10.21873/invivo.12395 PMC8193324 33910820

[B60] Qi R. Z. He S. L. Li Y. Zhao Y. W. Geng L. He J. (2023). Retrospective clinical study on integrated Chinese and western medicine in treatment of limited-stage small cell lung cancer. Chin. J. Integr. Med. 29 (8), 675–682. 10.1007/s11655-022-3682-9 36607585

[B61] Qi T. Li H. Li S. (2017). Indirubin improves antioxidant and anti-inflammatory functions in lipopolysaccharide-challenged mice. Oncotarget 8 (22), 36658–36663. 10.18632/oncotarget.17560 28525368 PMC5482685

[B62] Qian W. Cai X. Qian Q. Zhang W. Wang D. (2018). Astragaloside IV modulates TGF-β1-dependent epithelial-mesenchymal transition in bleomycin-induced pulmonary fibrosis. J. Cell Mol. Med. 22 (9), 4354–4365. 10.1111/jcmm.13725 29971947 PMC6111865

[B63] Qiu W. L. Hsu W. H. Tsao S. M. Tseng A. J. Lin Z. H. Hua W. J. (2021). WSG, a glucose-rich polysaccharide from Ganoderma lucidum, combined with cisplatin potentiates inhibition of lung cancer *in vitro* and *in vivo* . Polym. (Basel) 13 (24), 4353. 10.3390/polym13244353 PMC8705874 34960904

[B64] Qu L. Chen C. He W. Chen Y. Li Y. Wen Y. (2019). Glycyrrhizic acid ameliorates LPS-induced acute lung injury by regulating autophagy through the PI3K/AKT/mTOR pathway. Am. J. Transl. Res. 11 (4), 2042–2055. 31105816 PMC6511780

[B65] Ren M. Chen J. Xu H. Li W. Wang T. Chi Z. (2023). Ergolide covalently binds NLRP3 and inhibits NLRP3 inflammasome-mediated pyroptosis. Int. Immunopharmacol. 120, 110292. 10.1016/j.intimp.2023.110292 37182452

[B66] Shen J. Hua Z. Chai Y. (2022). Glycyrrhizic acid protects experimental sepsis rats against acute lung injury and inflammation. Evid. Based Complement. Altern. Med. 2022, 3571800, 3571800. 10.1155/2022/3571800 PMC9444394 36072408

[B67] Shen K. H. Hung J. H. Chang C. W. Weng Y. T. Wu M. J. Chen P. S. (2017). Solasodine inhibits invasion of human lung cancer cell through downregulation of miR-21 and MMPs expression. Chem. Biol. Interact. 268, 129–135. 10.1016/j.cbi.2017.03.005 28283413

[B68] Shen K. H. Hung J. H. Liao Y. C. Tsai S. T. Wu M. J. Chen P. S. (2020). Sinomenine inhibits migration and invasion of human lung cancer cell through downregulating expression of miR-21 and MMPs. Int. J. Mol. Sci. 21 (9), 3080. 10.3390/ijms21093080 32349289 PMC7247699

[B69] Shen M. Wang Y. J. Liu Z. H. Chen Y. W. Liang Q. K. Li Y. (2023). Inhibitory effect of Astragalus polysaccharide on premetastatic niche of lung cancer through the S1PR1-STAT3 signaling pathway. Evid. Based Complement. Altern. Med. 2023, 4010797, 4010797. 10.1155/2023/4010797 PMC9883101 36714534

[B70] Shi W. Hao J. Wu Y. Liu C. Shimizu K. Li R. (2022). Protective effects of heterophyllin B against bleomycin-induced pulmonary fibrosis in mice via AMPK activation. Eur. J. Pharmacol. 921, 174825, 174825. 10.1016/j.ejphar.2022.174825 35283110

[B71] Shi Z. (2013). Abstract 2245: overcoming cisplatin resistance by celastrol in cancer cells. Cancer Res. 73 (8), 2245. 10.1158/1538-7445.AM2013-2245

[B72] Su S. H. Sundhar N. Kuo W. W. Lai S. C. Kuo C. H. Ho T. J. (2022). Artemisia argyi extract induces apoptosis in human gemcitabine-resistant lung cancer cells via the PI3K/MAPK signaling pathway. J. Ethnopharmacol. 299, 115658. 10.1016/j.jep.2022.115658 36075273

[B73] Sui Y. Li S. Shi P. Wu Y. Li Y. Chen W. (2016). Ethyl acetate extract from Selaginella doederleinii Hieron inhibits the growth of human lung cancer cells A549 via caspase-dependent apoptosis pathway. J. Ethnopharmacol. 190, 261–271. 10.1016/j.jep.2016.06.029 27292193

[B74] Sun J. Zhang Q. Yang G. Li Y. Fu Y. Zheng Y. (2022). The licorice flavonoid isoliquiritigenin attenuates Mycobacterium tuberculosis-induced inflammation through Notch1/NF-κB and MAPK signaling pathways. J. Ethnopharmacol. 294, 115368. 10.1016/j.jep.2022.115368 35589023

[B75] Sun L. X. Li W. D. Lin Z. B. Duan X. S. Li X. F. Yang N. (2014). Protection against lung cancer patient plasma-induced lymphocyte suppression by Ganoderma lucidum polysaccharides. Cell Physiol. Biochem. 33 (2), 289–299. 10.1159/000356669 24525691

[B76] Tang J. C. Ren Y. G. Zhao J. Long F. Chen J. Y. Jiang Z. (2018). Shikonin enhances sensitization of gefitinib against wild-type EGFR non-small cell lung cancer via inhibition PKM2/stat3/cyclinD1 signal pathway. Life Sci. 204, 71–77. 10.1016/j.lfs.2018.05.012 29738778

[B77] Tímár J. Uhlyarik A. (2022). On-target side effects of targeted therapeutics of cancer. Pathol. Oncol. Res. 28, 1610694. 10.3389/pore.2022.1610694 36213163 PMC9537356

[B78] Wang H. Wu J. Fan H. Ji Y. Han C. Li C. (2022). The impact of catalpol on proliferation, apoptosis, migration, and oxidative stress of lung cancer cells based on Nrf2/ARE signaling. Biomed. Res. Int. 2022, 5621341, 5621341. 10.1155/2022/5621341 35898682 PMC9313965

[B79] Wang J. Wu Q. Ding L. Song S. Li Y. Shi L. (2021). Therapeutic effects and molecular mechanisms of bioactive compounds against respiratory diseases: traditional Chinese medicine theory and high-frequency use. Front. Pharmacol. 12, 734450. 10.3389/fphar.2021.734450 34512360 PMC8429615

[B80] Wang N. Tan H. Y. Chan Y. T. Guo W. Li S. Feng Y. (2017). Identification of WT1 as determinant of heptatocellular carcinoma and its inhibition by Chinese herbal medicine Salvia chinensis Benth and its active ingredient protocatechualdehyde. Oncotarget 8 (62), 105848–105859. 10.18632/oncotarget.22406 29285297 PMC5739684

[B81] Waqar S. N. Morgensztern D. (2017). Treatment advances in small cell lung cancer (SCLC). Pharmacol. Ther. 180, 16–23. 10.1016/j.pharmthera.2017.06.002 28579387

[B82] Wei C. Yao X. Jiang Z. Wang Y. Zhang D. Chen X. (2019). Cordycepin inhibits drug-resistance non-small cell lung cancer progression by activating AMPK signaling pathway. Pharmacol. Res. 144, 79–89. 10.1016/j.phrs.2019.03.011 30974169

[B83] Wu C. Zhuang Y. Jiang S. Tian F. Teng Y. Chen X. (2017). Cinnamaldehyde induces apoptosis and reverses epithelial-mesenchymal transition through inhibition of Wnt/β-catenin pathway in non-small cell lung cancer. Int. J. Biochem. Cell Biol. 84, 58–74. 10.1016/j.biocel.2017.01.005 28093328

[B84] Wu C. Y. Yang Y. H. Lin Y. S. Chang G. H. Tsai M. S. Hsu C. M. (2021). Dihydroisotanshinone I induced ferroptosis and apoptosis of lung cancer cells. Biomed. Pharmacother. 139, 111585. 10.1016/j.biopha.2021.111585 33862493

[B85] Wu L. Liu T. Xiao Y. Li X. Zhu Y. Zhao Y. (2016). Polygonatum odoratum lectin induces apoptosis and autophagy by regulation of microRNA-1290 and microRNA-15a-3p in human lung adenocarcinoma A549 cells. Int. J. Biol. Macromol. 85, 217–226. 10.1016/j.ijbiomac.2015.11.014 26562549

[B86] Wu T. H. Yeh K. Y. Wang C. H. Wang H. Li T. L. Chan Y. L. (2019). The combination of Astragalus membranaceus and Angelica sinensis inhibits lung cancer and cachexia through its immunomodulatory function. J. Oncol. 2019, 9206951. 10.1155/2019/9206951 31781219 PMC6875282

[B87] Wu X. Wang W. Chen Y. Liu X. Wang J. Qin X. (2018). Glycyrrhizin suppresses the growth of human NSCLC cell line HCC827 by downregulating HMGB1 level. Biomed. Res. Int. 2018, 6916797. 10.1155/2018/6916797 29568761 PMC5820661

[B88] Xi P. Niu Y. Zhang Y. Li W. Gao F. Gu W. (2022). The mechanism of dioscin preventing lung cancer based on network pharmacology and experimental validation. J. Ethnopharmacol., 292, 115138. 10.1016/j.jep.2022.115138 35245631

[B89] Xie H. J. Zhao J. Zhuo-Ma D. Zhan-Dui N. Er-Bu A. Tsering T. (2019). Inhibiting tumour metastasis by DQA modified paclitaxel plus ligustrazine micelles in treatment of non-small-cell lung cancer. Artif. Cells Nanomed Biotechnol. 47 (1), 3465–3477. 10.1080/21691401.2019.1653900 31432702

[B90] Xu L. Meng X. Xu N. Fu W. Tan H. Zhang L. (2018). Gambogenic acid inhibits fibroblast growth factor receptor signaling pathway in erlotinib-resistant non-small-cell lung cancer and suppresses patient-derived xenograft growth. Cell Death Dis. 9 (3), 262. 10.1038/s41419-018-0314-6 29449529 PMC5833807

[B91] Xu X. Q. Deng W. Q. Wang D. Y. Li M. Kou D. L. Zhang P. T. (2021). Chinese medicine treatment prolonged survival in small cell lung cancer patients: a clinical observation. Chin. J. Integr. Med. 27 (7), 496–501. 10.1007/s11655-020-3197-1 32500318

[B92] Xu Z. Mei J. Tan Y. (2017). Baicalin attenuates DDP (cisplatin) resistance in lung cancer by downregulating MARK2 and p-Akt. Int. J. Oncol. 50 (1), 93–100. 10.3892/ijo.2016.3768 27878245

[B93] Xueting C. Jie Y. Chunping H. U. Peng C. (2017). Dihydroartemisinin enhances the sensitivity of gefitinib in non-small cell lung cancer cells by inhibiting STAT3. Chin. Sci. Bull. 62 (18), 2013–2019. 10.1360/N972017-00204

[B94] Yang H. Tong Z. Shen L. Sun Y. U. Hoffman R. M. Huang J. (2020). Brucea javanica increases survival and enhances gemcitabine efficacy in a patient-derived orthotopic xenograft (PDOX) mouse model of pancreatic cancer. Anticancer Res. 40 (9), 4969–4978. 10.21873/anticanres.14500 32878785

[B95] Ye C. Zhang N. Zhao Q. Xie X. Li X. Zhu H. P. (2021). Evodiamine alleviates lipopolysaccharide-induced pulmonary inflammation and fibrosis by activating apelin pathway. Phytother. Res. 35 (6), 3406–3417. 10.1002/ptr.7062 33657655

[B96] Ye Y. T. Zhong W. Sun P. Wang D. Wang C. Hu L. M. (2017). Apoptosis induced by the methanol extract of Salvia miltiorrhiza Bunge in non-small cell lung cancer through PTEN-mediated inhibition of PI3K/Akt pathway. J. Ethnopharmacol. 200, 107–116. 10.1016/j.jep.2016.12.051 28088493

[B97] Ying Y. Sun C. B. Zhang S. Q. Chen B. J. Yu J. Z. Liu F. Y. (2021). Induction of autophagy via the TLR4/NF-κB signaling pathway by astragaloside Ⅳ contributes to the amelioration of inflammation in RAW264.7 cells. Biomed. Pharmacother. 137, 111271. 10.1016/j.biopha.2021.111271 33561643

[B98] You C. Sun Y. Zhang S. Tang G. Zhang N. Li C. (2018). Trichosanthin enhances sensitivity of non-small cell lung cancer (NSCLC) TRAIL-resistance cells. Int. J. Biol. Sci. 14 (2), 217–227. 10.7150/ijbs.22811 29483839 PMC5821042

[B99] You X. Y. Xue Q. Fang Y. Liu Q. Zhang C. F. Zhao C. (2015). Preventive effects of Ecliptae Herba extract and its component, ecliptasaponin A, on bleomycin-induced pulmonary fibrosis in mice. J. Ethnopharmacol. 175, 172–180. 10.1016/j.jep.2015.08.034 26385580

[B100] Yuan J. Y. Tong Z. Y. Dong Y. C. Zhao J. Y. Shang Y. (2022). Research progress on icariin, a traditional Chinese medicine extract, in the treatment of asthma. Allergol. Immunopathol. Madr. 50 (1), 9–16. 10.15586/aei.v50i1.490 34873891

[B101] Zhang J. L. Huang W. M. Zeng Q. Y. (2015). Atractylenolide I protects mice from lipopolysaccharide-induced acute lung injury. Eur. J. Pharmacol. 765, 94–99. 10.1016/j.ejphar.2015.08.022 26297303

[B102] Zhang Q. Zhu S. Cheng X. Lu C. Tao W. Zhang Y. (2018). Euphorbia factor L2 alleviates lipopolysaccharide-induced acute lung injury and inflammation in mice through the suppression of NF-κB activation. Biochem. Pharmacol. 155, 444–454. 10.1016/j.bcp.2018.07.025 30055150

[B103] Zhang Y. Wang J. Hui B. Sun W. Li B. Shi F. (2019). Pristimerin enhances the effect of cisplatin by inhibiting the miR-23a/Akt/GSK3β signaling pathway and suppressing autophagy in lung cancer cells. Int. J. Mol. Med. 43 (3), 1382–1394. 10.3892/ijmm.2019.4057 30664149 PMC6365073

[B104] Zhao X. Y. Yang Y. Y. Feng J. L. Feng C. L. (2023). Network pharmacology prediction and experimental validation of trichosanthes-fritillaria thunbergii action mechanism against lung adenocarcinoma. J. Vis. Exp. 10.3791/64847 36939253

[B105] Zhu J. Huang R. Yang R. Xiao Y. Yan J. Zheng C. (2021). Licorice extract inhibits growth of non-small cell lung cancer by down-regulating CDK4-Cyclin D1 complex and increasing CD8^+^ T cell infiltration. Cancer Cell Int. 21 (1), 529. 10.1186/s12935-021-02223-0 34641869 PMC8507331

[B106] Zhu J. Wang H. Chen F. Lv H. Xu Z. Fu J. (2018). Triptolide enhances chemotherapeutic efficacy of antitumor drugs in non-small-cell lung cancer cells by inhibiting Nrf2-ARE activity. Toxicol. Appl. Pharmacol. 358, 1–9. 10.1016/j.taap.2018.09.004 30196066

[B107] Zhu Y. Chai Y. Xiao G. Liu Y. Xie X. Xiao W. (2022). Astragalus and its formulas as a therapeutic option for fibrotic diseases: pharmacology and mechanisms. Front. Pharmacol. 13, 1040350. 10.3389/fphar.2022.1040350 36408254 PMC9669388

[B108] Zou X. Huang Z. Zhan Z. Yuan M. Zhang Y. Liu T. (2023). The alcohol extracts of Sceptridium ternatum (Thunb.) Lyon exert anti-pulmonary fibrosis effect through targeting SETDB1/STAT3/p-STAT3 signaling. J. Ethnopharmacol. 313, 116520. 10.1016/j.jep.2023.116520 37120058

[B109] Zraik I. M. Heß-Busch Y. (2021). Management von Nebenwirkungen der Chemotherapie und deren Langzeitfolgen [Management of chemotherapy side effects and their long-term sequelae]. Urol. A 60 (7), 862–871. 10.1007/s00120-021-01569-7 34185118

